# Exploration of the COVID-19 pandemic at the neighborhood level in an intra-urban setting

**DOI:** 10.3389/fpubh.2023.1128452

**Published:** 2023-04-13

**Authors:** Tillman Schmitz, Tobia Lakes, Georgianna Manafa, Christoph Lambio, Jeffrey Butler, Alexandra Roth, Nicolai Savaskan

**Affiliations:** ^1^Applied Geoinformation Science, Geography Department, Humboldt University Berlin, Berlin, Germany; ^2^Integrative Research Institute on Transformations of Human Environment Systems (IRI THESys), Berlin, Germany; ^3^Department of Public Health Neukölln, District Office Neukölln, Berlin, Germany

**Keywords:** COVID-19, spatiotemporal analysis, public health measure, spatial scan statistics, age groups, urban health

## Abstract

The COVID-19 pandemic represents a worldwide threat to health. Since its onset in 2019, the pandemic has proceeded in different phases, which have been shaped by a complex set of influencing factors, including public health and social measures, the emergence of new virus variants, and seasonality. Understanding the development of COVID-19 incidence and its spatiotemporal patterns at a neighborhood level is crucial for local health authorities to identify high-risk areas and develop tailored mitigation strategies. However, analyses at the neighborhood level are scarce and mostly limited to specific phases of the pandemic. The aim of this study was to explore the development of COVID-19 incidence and spatiotemporal patterns of incidence at a neighborhood scale in an intra-urban setting over several pandemic phases (March 2020–December 2021). We used reported COVID-19 case data from the health department of the district Berlin-Neukölln, Germany, additional socio-demographic data, and text documents and materials on implemented public health and social measures. We examined incidence over time in the context of the measures and other influencing factors, with a particular focus on age groups. We used incidence maps and spatial scan statistics to reveal changing spatiotemporal patterns. Our results show that several factors may have influenced the development of COVID-19 incidence. In particular, the far-reaching measures for contact reduction showed a substantial impact on incidence in Neukölln. We observed several age group-specific effects: school closures had an effect on incidence in the younger population (< 18 years), whereas the start of the vaccination campaign had an impact primarily on incidence among the elderly (> 65  years). The spatial analysis revealed that high-risk areas were heterogeneously distributed across the district. The location of high-risk areas also changed across the pandemic phases. In this study, existing intra-urban studies were supplemented by our investigation of the course of the pandemic and the underlying processes at a small scale over a long period of time. Our findings provide new insights for public health authorities, community planners, and policymakers about the spatiotemporal development of the COVID-19 pandemic at the neighborhood level. These insights are crucial for guiding decision-makers in implementing mitigation strategies.

## 1. Introduction

The emergence of the COVID-19 pandemic has had severe social and economic consequences for societies worldwide. More than 620 million cases have been reported globally, with approximately 6.5 million deaths from the beginning of the pandemic in December 2019 until November 2022 (1). The pandemic has proceeded in different phases, the development of case numbers in each phase in a specific location being determined by several factors that have constantly changed and interacted (2), such as the implementation of public health and social measures (2, 3), the emergence of new virus variants of concern (VOCs) (4), the immunity of the population (either by vaccine or infection) (5, 6), behavioral factors (7, 8), socioeconomic factors of the population (9), and seasonality (10, 11). In particular, the implementation and effectiveness of measures at regional or local level can differ considerably, leading to spatial differences in the incidence of COVID-19 (12–14).

The effectiveness of the various measures implemented to reduce the spread of COVID-19 has been critically discussed, owing to their negative outcomes on the economic, social, and demographic status of the population, notably the intensification of existing inequalities (15). Measures comprise actions taken by individuals, institutions, communities, and local and national governments, such as non-pharmaceutical interventions, physical distancing measures, pre- and post-exposure prophylaxis, and vaccines (16). Many countries have called for better preparedness for pandemics (17). The decision to implement measures to control any infectious diseases relies on functioning disease surveillance, which involves the continuous, systematic collection, analysis, and interpretation of disease outbreaks and their related factors (18). During the COVID-19 pandemic, it became particularly apparent that timely, high-quality, accessible, and detailed data are needed to help decision-makers rationally develop mitigation strategies and allocate resources (19–21).

The provision of publicly available COVID-19 datasets has led to an increasing number of studies that explored and analyzed spatiotemporal patterns of COVID-19 incidence and its underlying geographic factors (22, 23). These studies in the field of descriptive epidemiology, which “provides a way of organizing and analyzing these data to describe the variations in disease frequency among populations by geographical areas and over time (i.e., person, place, and time),” can play an important role in “generat[ing] hypotheses of etiologic research” (24). Intra-urban COVID-19 studies at the zip code or census tract level revealed that incidences exhibit heterogeneous spatial distributions, with changing patterns between single pandemic phases (25–27). The detection and monitoring of disease clusters, i.e., the aggregations of relatively uncommon events or diseases in space and/or time (28), have widely been used to provide important information for disease surveillance and for improving the understanding of the spread and risk of COVID-19 (29, 30). The identification of disease clusters in space has drawn attention to specific contextual settings, including the socioeconomic, environmental, or demographic characteristics of an area (31–33), while temporal disease cluster analyses indicate how interventions may have influenced infection dynamics (34, 35).

Studies have shown that the spread of COVID-19 is strongly influenced by socioeconomic structures in the population, socioeconomically disadvantaged areas being more severely affected (36, 37). Besides studies analyzing socioeconomic factors, only a few studies have specifically examined the age distribution of the infected population over time (38, 39). For Germany, for example, it has been shown that the incidence among age groups differed within each pandemic phase and that age group-specific incidence changed over time (40). Understanding this phenomenon is crucial for the development of mitigation strategies and thus the protection of vulnerable population groups (39, 41, 42).

However, detailed research on COVID-19 incidence at the neighborhood level remains scarce, owing to the lack of available COVID-19 data and information on influencing factors (23). Thus, changes in incidence in different neighborhoods over the different pandemic phases remain largely unexplored. Further, little attention has been paid so far to how measures to control the spread of the disease have influenced incidence and demographic characteristics on a small scale.

We therefore aimed (1) to explore the development of COVID-19 incidence over time in relation to the implemented measures, (2) to examine age group-specific incidence over time, and (3) to identify changes in spatial patterns and clusters of COVID-19 incidence over several pandemic phases. We chose the district Berlin-Neukölln, Germany, as a case study. In contrast to other studies, which tend to focus on larger spatial scales and shorter time frames, this cross-sectional study demonstrates the usefulness of investigating the course of the pandemic and the underlying processes at the neighborhood level over a longer period of time. Continuous data exploration on a small scale can undoubtedly serve as a basis for further detailed investigations of these issues. We conducted a descriptive epidemiological study, involving spatiotemporal exploration to identify high-risk areas across the different phases of the COVID-19 pandemic. The study thus demonstrates the importance of analyzing person-, place-, and time-related data for developing measures that target vulnerable locations and groups in a timely manner. This knowledge is essential not only for this pandemic, but, perhaps even more important, for future pandemics. Additionally, the findings are decisive for identifying underlying inequalities in local health settings and interventions.

## 2. Materials and methods

### 2.1. Study area

In line with our research aims, we used reported COVID-19 case data at the neighborhood level for the Neukölln district in Berlin. This case study represented a good candidate for such a detailed analysis because of the high temporal and spatial dynamics in the reported COVID-19 cases and the very diverse characteristics of the district in terms of population and urban structure.

**Figure 1:**
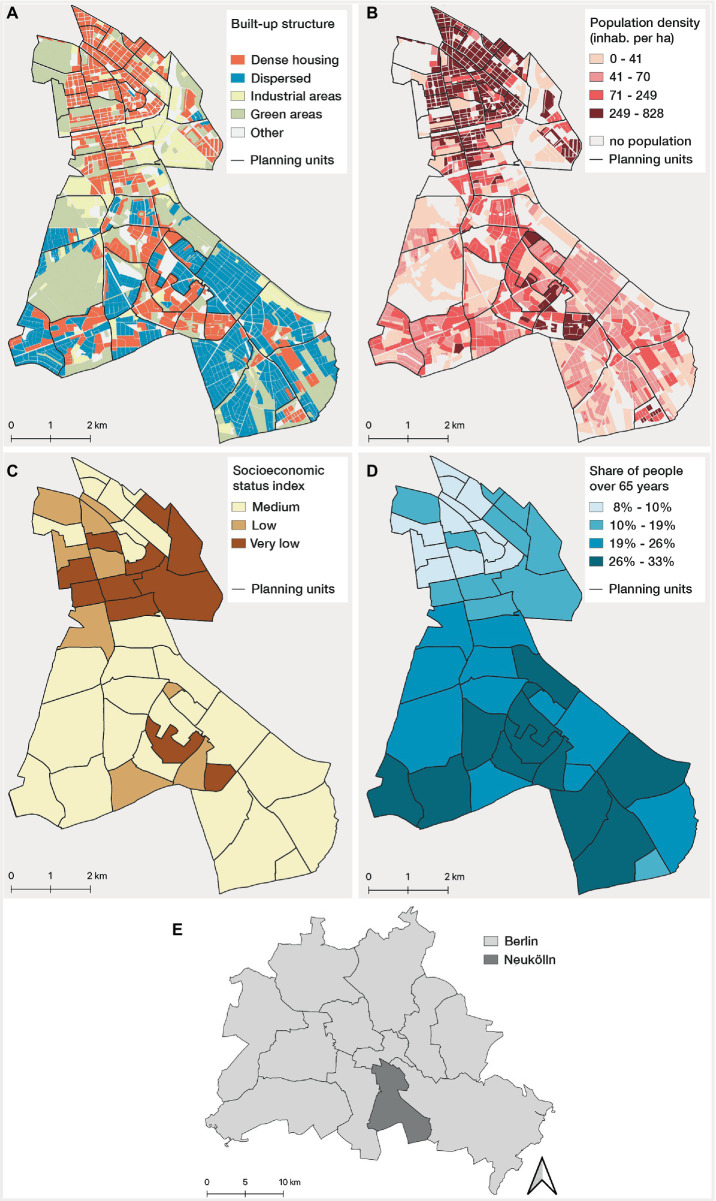
Built-up and demographic characteristics of Neukölln: **(A)** built-up structures at block level*, and **(B)** population density at block level*, **(C)** socioeconomic status index** (calculated on the basis of unemployment rate, child poverty and people receiving social benefits), and **(D)** share of people >65 years old at planning unit level; **(E)** location of Neukölln in Berlin. Data source: Senate for Building, Housing and Transport, Berlin (44) and Statistics Office of Berlin-Brandenburg (43). *The block level is the smallest subdivision of the Berlin urban area in the regional reference system (43). **There is also the class “high” in Berlin. But no planning units are classified as “high” in Berlin-Neukölln.

Neukölln, one of the 12 districts in Berlin, is located in the southern part of the capital city of Germany (Figure 1). With a population of 327,100 inhabitants in an area of 44.9 km^2^, Neukölln was, in 2021, the third most densely populated district in Berlin (7,284 persons/km^2^) and, in terms of population, the 20th largest city in Germany (43). The district exhibits distinct spatial differences in regard to built-up structures and environmental features as well as the socioeconomic and demographic characteristics of the population (44) (Figures 1A–D). The greater part of the north has a high population density, with residential buildings primarily consisting of dense housing structures (e.g., multi-story block developments and social housing estates; Figures 1A,B). Several areas in the north register low to very low on the index of socioeconomically disadvantaged areas (Figure 1C). The district has a rather young population with a low proportion of elderly people (Figure 1D). In contrast, large parts of the southern district are dominated by more dispersed housing structures with primarily single-family or semi-detached houses, resulting in a comparatively low population density. In the center of the southern district, however, there is a sub-district that differs from its surroundings, having denser housing structures and a higher population density. Most areas of the south register medium on the socioeconomic status index, whereas the area in the southern center registers low to very low on the index. The population is considerably older in the south than in the north of the district, having many areas where those over 65 years make up nearly one-third of the population. As a unit of analysis, we used the 46 planning units of the so-called small Lebenswelt-oriented areas (‘lebensweltlich orientierte Räume’), which were created by the city of Berlin as relatively homogeneous spatial entities within a district as a basis for the planning and prediction of demographic and socioeconomic development (45).

### 2.2. Data

#### 2.2.1. COVID-19 data

We used reported PCR test-confirmed COVID-19 case data provided by the Neukölln department of health for the period from 1 March 2020 to 26 December 2021. We focused on this time period because, from the beginning of 2022, positive rapid antigen test results from test centers were also legally accepted and thus recorded as cases in Germany, which tended to bias the data and reduce their quality. In addition, the predominant Omicron variant (B.1.1.529) in 2022 proved almost ubiquitous. The dataset contained cases that were attributed by information on the report date (date when the positive test result was added to the local health department’s database), sex, age, and planning unit in which the residential address of the infected person was located. We omitted cases found in facilities such as nursing homes and refugee shelters (in total 960 cases) to avoid biases in the COVID-19 case distribution, since the risk of infection is particularly high in these facilities, and the facilities are not evenly distributed across Neukölln. After additional data cleaning (removal of duplicates, data with missing age or spatial information), a total of 37,600 cases remained for our study period.

We then classified each case into one of the six pandemic phases (four infection waves and two summer plateaus), following the classification of the national public health institute of Germany (Robert Koch Institute, RKI) (46). We then calculated, at the district level, weekly crude incidence rates per 100,000 population (number of new cases divided by total population) and weekly age group-specific incidence rates per 100,000 population (see explanation of demographic data below). For our spatiotemporal analysis, we calculated the cumulative incidence rates per 100,000 population aggregated at the planning unit level for each of the six pandemic phases.

#### 2.2.2. Population data

We explored population data for the planning units for the first half of 2021 obtained from the Statistics Office of Berlin-Brandenburg (43), which showed that the data have not changed markedly during the course of the pandemic, so we assumed that the one point in time was valid for the overall period. We used the total population and the age information for classifying the population into the following age groups: (1) 0–18 years old [children and young adolescents, who have been particularly affected by state-imposed restrictions (47)], (2) 19–44 years old and 45–64 years old (working population), and (3) > 65 years old [the elderly being the most vulnerable population (48)]. This followed earlier studies that have shown varying effects of the implementation of interventions on COVID-19 development between these age groups (39, 41, 49).

### 2.3. Methods

#### 2.3.1. Development of COVID-19 incidence and influencing factors

To understand the development of COVID-19 in the whole study area over time, we created time graphs from early March 2020 to late December 2021 and described the implemented measures in each of the six pandemic phases. To identify the implemented measures, we combined information from a web-data platform (50), expert knowledge of the Neukölln department of health, and published COVID-19 regulations from the Berlin Senate (i.e., state government in Berlin) (51). Since measures most often came in packages, it was often impossible to evaluate the relationship between single measures and their impact on COVID-19 incidence. Therefore, we focused our exploration on measures that had already been proven to be influential on COVID-19 incidence (2, 3, 52). These measures included social and physical distancing (e.g., restrictions on personal contacts, closure of public places and schools, cancelation of public mass gatherings), mask-wearing requirements, specific test strategies, and vaccination campaigns. We identified the date when measures were implemented by consulting the COVID-19 regulations published by the Berlin Senate. To complement our analysis, we used an existing measure-index to approximate the stringency and duration of measures (53) (see also Supplementary Figure 1 for detailed explanations). By plotting this index over time, we were able to visually identify far-reaching measures through particularly strong index changes.

In addition, we identified other potentially associated factors that were known either from earlier studies or from the expert knowledge of the Neukölln department of health, such as differences in virus variants, school holidays, and Neukölln-specific events that might explain major short-term incidence changes.

#### 2.3.2. Spatiotemporal pattern analysis

To assess spatiotemporal patterns at the neighborhood level, we first visualized the COVID-19 incidence for each of the six phases in choropleth maps. We used a five-quantile categorization to assure an equal number of planning units in each category and to allow for comparability across different phases (54).

We then identified spatial clusters of planning units with high or low relative risk of COVID-19 for each pandemic phase, i.e., we compared whether more or fewer observed than expected cases were detected within a certain area relative to randomly distributed cases. In this study, we used a discrete Poisson spatial scan statistic (55), which was developed to detect statistically significant disease clusters and has already been successfully applied for COVID-19 research on a small scale (25, 32, 33, 35). The scan statistic is defined by circular windows located around the centroids of each planning unit, each window representing a potential candidate cluster. For each candidate cluster, the number of observed cases was compared with the number of expected cases, calculated by a Poisson distribution. A maximum likelihood test was performed under the null hypothesis that there was no difference in the risk of COVID-19 between the inside and the outside of the window. Clusters with increased risk were reported if the likelihood ratio was greater than 1. A significance test *via* Monte Carlo simulations (999 replications) evaluated whether more observed than expected cases were detected within the window relative to randomly distributed cases over space. We compared different maximum spatial cluster sizes and then iteratively decided on the maximum spatial cluster size of 7.5% of the population at risk to avoid large clusters and concentrate on small clusters (56). We also computed the relative risk of a cluster, which is the risk within a cluster divided by the risk outside. For example, a relative risk value of 2.5 means that a population within that location is 2.5 times more likely to be exposed to COVID-19 than in other locations. Thus, we identified statistically significant clusters (*p* ≤ 0.05) with high and low relative risks for each of the six pandemic phases (see also Supplementary Table 1 for detailed explanations of the used spatial scan statistic parameter settings).

Finally, we implemented a web-based visualization tool to allow for a more detailed interactive exploration of the data and our findings.^1^ The tool is written in R (57) and leverages the R Shiny library (58).

## 3. Results

### 3.1. Development of incidence in Neukölln

**Figure 2:**
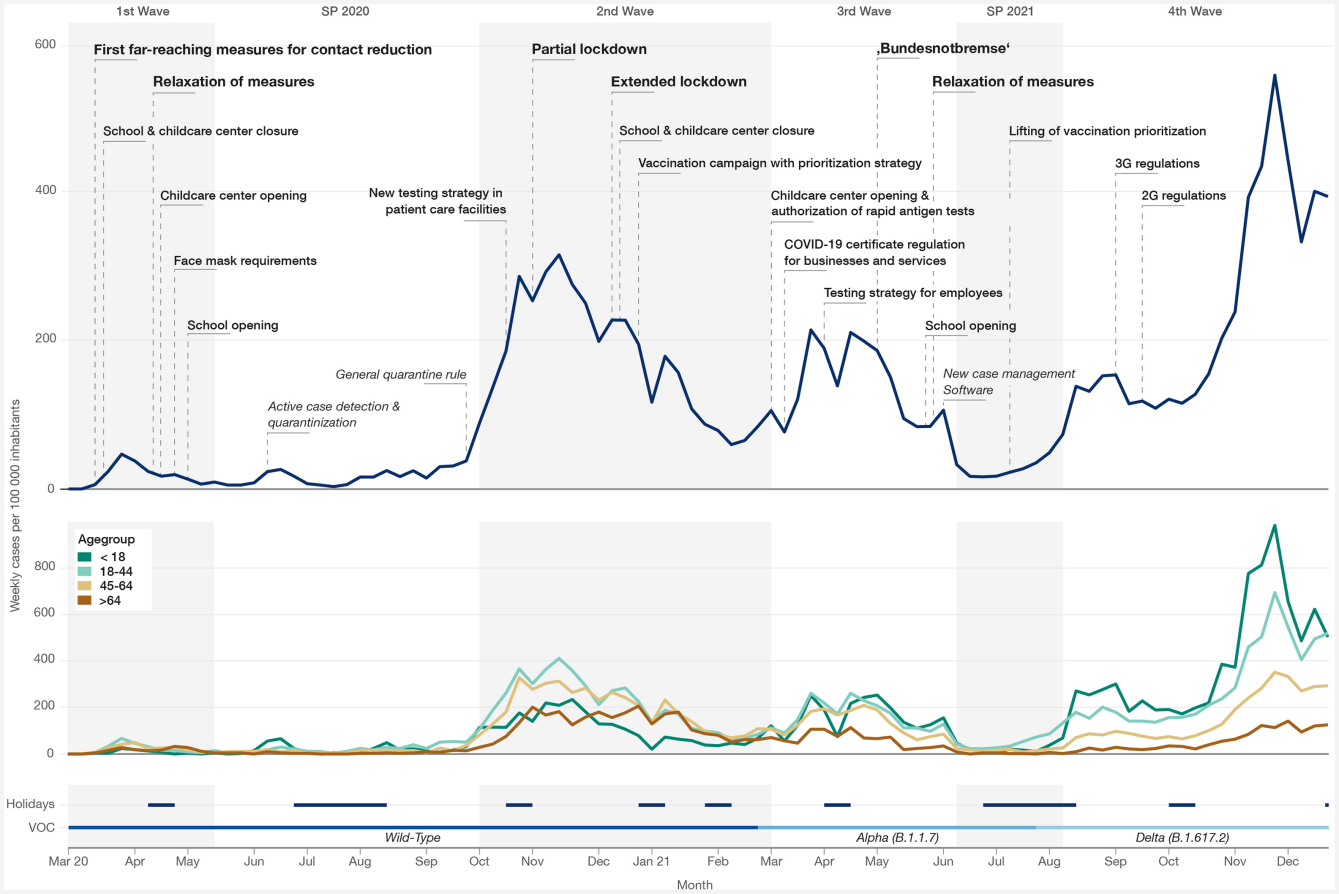
Development of incidence across pandemic phases, including the four waves and two summer plateaus (SP) during the study period. Top: incidence in Neukölln over time with far-reaching public health and social measures (bold), distinct measures/influencing factors (regular), and distinct measures/influencing factors in Neukölln (italic); middle: age group-specific incidence; bottom: school holidays and variant of concern (VOC). Detailed descriptions of each measure can be found in Supplementary Table 2.

For the investigated time period from the first positive cases in early March until the end of the fourth wave (late December 2021), we observed a highly dynamic development of the overall and age group-specific incidence for the Neukölln district (Figure 2). We identified a large number of measures that were implemented during this time. Figure 2 depicts only a subset of implemented measures and influencing factors (for detailed descriptions see Supplementary Table 2). Further details on Berlin-wide COVID-19 regulations and the measure-index are presented in Supplementary Figure 1.

The first cases in Neukölln were recorded in early March 2020, 1 month after the virus was reported in Germany. From then on, incidence increased in Neukölln and reached its first peak in mid-March 2020. During this time, far-reaching measures for contact reduction were put in place (see Figure 2). These measures included the closure of schools, childcare centers, businesses, institutions, and services, plus additional restrictions on public events and private gatherings. Shortly thereafter, a lockdown was declared to reduce contact, which added further restrictions. The measures succeeded in decreasing incidence in Neukölln in the following weeks. At the end of April 2020, the measures were eased for the first time, allowing schools, childcare centers, and businesses to open again under specific hygiene conditions. Incidence remained mainly low in Neukölln during the summer months, with one exception in mid-June 2020 when incidence rose to a sharp peak owing to a large outbreak of cases in a residential complex in the north of the district. Measures within the residential complex, such as large-scale testing and strict quarantine rules, were implemented by the Neukölln department of health to prevent any further spread of the illness.

The second wave started at the end of September 2020, with a steep rise in incidence until a temporary peak in mid-November 2020. Around that time, a partial lockdown was introduced to control the spread of the virus. Businesses, institutions, and services had to close, and restrictions were imposed on public events and private gatherings. However, the measures did not achieve the desired effect, leading to an extended lockdown in mid-December 2020, when schools and childcare centers were closed, and enhanced restrictions were imposed on private gatherings. Although a decrease in incidence was already evident in Neukölln after the introduction of the partial lockdown, the extended lockdown ultimately contributed to a more significant easing of the situation. Incidence started to decrease, reaching a temporary low during the Christmas break in 2020, followed by a short period of increase during the first weeks of January 2021 and a further fall, with substantially fewer case numbers in mid-February 2021. The vaccination campaign, which focused initially on people over 80 as well as people working in medical facilities and nursing staff, also started during the second wave.

The third pandemic wave followed directly on from the second wave without any relaxation of measures. The more contagious virus variant Alpha (B.1.1.7) became the dominant virus variant in Berlin at the end of February 2021, resulting in a large increase in case numbers until the end of March 2021. In March, rapid antigen tests became available, which led to the opening of many centers where persons could get tested once a week free of charge. During this time, test-related access requirements for businesses and services were also introduced for the first time and then gradually extended over the following weeks. The Easter holidays at the beginning of April 2021 led to a temporary drop in incidence, followed, however, by a rise that lasted until mid-April. Rising case numbers could not be controlled by the measures already in place, which resulted in an expansion of measures at the end of April 2021 (“Bundesnotbremse,” literally “federal emergency brake”). The measures were connected for the first time with the numbers of regional cases. At the time the new regulation took effect, for Berlin as a state, the measures included the reduction of contact in private and business-related settings and the closure of schools and childcare centers. The result was a fall to a low level of cases by the end of June 2021. The data show a temporary peak in early June 2021, which may be attributed to a change of the software used by the Neukölln department of health to record cases. Many cases had to be updated in the new software, resulting in a brief time lag of new cases and thus leading to an apparent temporary increase in numbers. The decrease in incidence continued in Neukölln after the short peak in June 2021. Already by early June, however, interventions were partly revoked for Berlin, which permitted the opening of schools and fewer restrictions on private gatherings.

However, the trend of low incidence did not last long during the summer months of 2021, and it began to rise rapidly again in July, owing to the new Delta (B.1.617.2) variant, which had become the most dominant virus variant in Berlin by then. This new and very contagious virus variant led to an unprecedented surge in cases in a short period of time. In Neukölln, a small, wave-like trend was visible from August until October. During that time, new regulations for access restrictions were implemented, meaning that persons needed to be vaccinated, recovered, or tested negative in order to access businesses, institutions, and services. Shortly thereafter, this regulation was tightened, and access was allowed only to vaccinated and recovered persons. Nevertheless, from mid-October 2021 onward, incidence again increased strongly, reaching a peak at the end of November. From the beginning of December, a sharp drop occurred, with another small peak 2 weeks before Christmas 2021. Although there has been an unprecedented increase in cases, only a few of the social and physical distancing measures were implemented during the fourth pandemic wave.

### 3.2. Development of age group-specific incidence

The first wave was characterized by high incidence among the working population (18–44 and 45–65 years), whereas the younger population (< 18 years) exhibited a very low incidence (Table 1). Incidence across all age groups decreased similarly after the implementation of the first set of measures (Figure 2). With the first easing of measures at the end of April 2020, the elderly (>65 years) were the most affected age group for a short time. During the summer months, incidence among the elderly decreased again and remained at a low level. In contrast, the young population, who had hitherto been the least affected, showed two short-term increases, one related to the outbreak of cases in a residential complex and the other occurring shortly after the summer holidays.

**Table 1:** Summary statistics of COVID-19 cases across age groups in Neukölln by pandemic phase.

Age groups	Wave 1 (*n* = 675)		SP 2020 (*n* = 1,056)		Wave 2 (*n* = 12,668)		Wave 3 (*n* = 6,534)		SP 2021 (*n* = 608)		Wave 4 (*n* = 16,059)		Total cases	Incidence per 100 k	Total cases	Incidence per 100 k	Total cases	Incidence per 100 k	Total cases	Incidence per 100 k	Total cases	Incidence Per 100 k	Total cases	Incidence Per 100 k
0–17	43 (6.4%)	80.8	195 (18.5%)	366.2	1,278 (10.1%)	2400.3	1,230 (18.8%)	2310.1	67 (11.0%)	125.8	4,354 (27.1%)	8177.4
18–44	336 (49.8%)	246.5	600 (56.8%)	440.2	6,368 (50.3%)	4672.1	3,241 (49.6%)	2377.9	438 (72.0%)	321.4	8,345 (52.0%)	6122.6
45–64	194 (28.7%)	240.9	204 (19.3%)	253.3	3,414 (26.9%)	4239.7	1,542 (23.6%)	1914.9	91 (15.0%)	113.0	2,663 (16.6%)	3307.0
> 65	102 (15.1%)	176.2	57 (5.4%)	98.5	1,608 (12.7%)	2778.3	521 (8.0%)	900.2	12 (2.0%)	20.7	697 (4.3%)	1204.3
Age median (years)	41		32		38		34		32		31	

SP, summer plateau.

At the beginning of the second wave, incidence increased across all age groups, the most rapid increment and highest overall incidence being among the age group 18–44. Incidence among the elderly and the young was relatively low compared to the other age groups until mid-November 2020. However, after the implementation of measures in late November, the rate among the elderly increased, while it decreased steadily among the young, such that the elderly were much more affected than the young by the end of the year. Toward the end of the second wave, incidence among all age groups was back at a similar level.

The beginning of the third wave was characterized by a marked increase in cases among the young and working population. In contrast, incidence among the elderly remained low. A temporary decrease was observed in all age groups over the Easter holidays, the most significant decline being among the young. However, the rate increased again shortly thereafter across all age groups. Compared to the previous waves, the younger population was by then much more affected than the elderly. Again, the implementation of extensive control measures in April 2021 led to a decline in cases across all age groups. During the summer plateau of 2021, the incidence was similar across age groups, whereas, from early July 2021 onward, the incidence in the 18–44 age group again rose sharply.

With the emergence of the Delta (B.1.617.2) variant, the age group-specific incidence underwent a drastic change. Incidence among the young population rose sharply, remaining highest among all age groups over the entire fourth wave, although the pattern was similar among adults aged 18–44, whereas the incidence among those aged 45–64 and the elderly was comparatively low until the end of 2021.

### 3.3. Spatiotemporal pattern analysis

**Figure 3:**
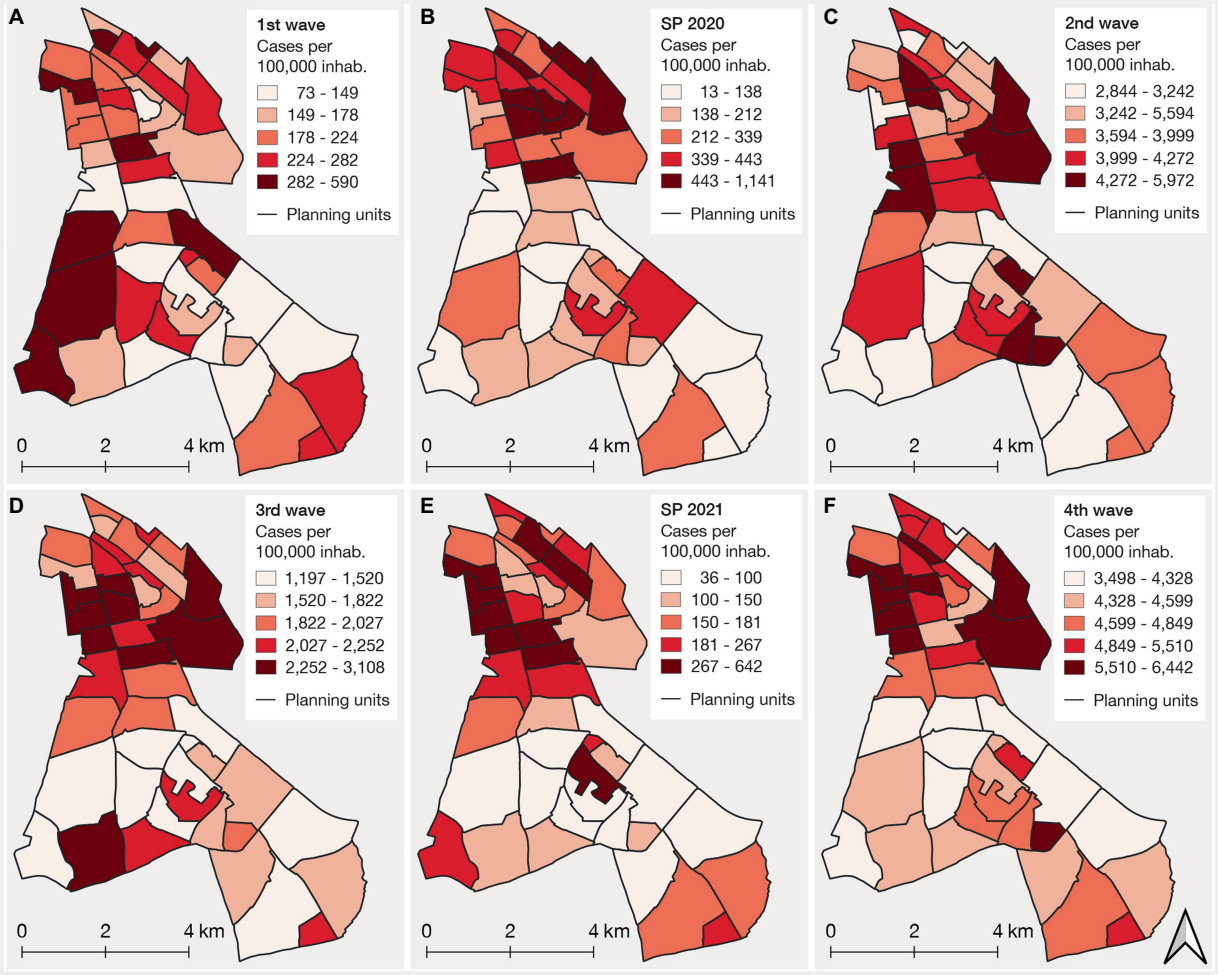
Cumulative incidence rate maps across the six pandemic phases, including the four waves **(A,C,D,F)** and two summer plateaus (SP) **(B,E)** during the study period.

**Figure 4:**
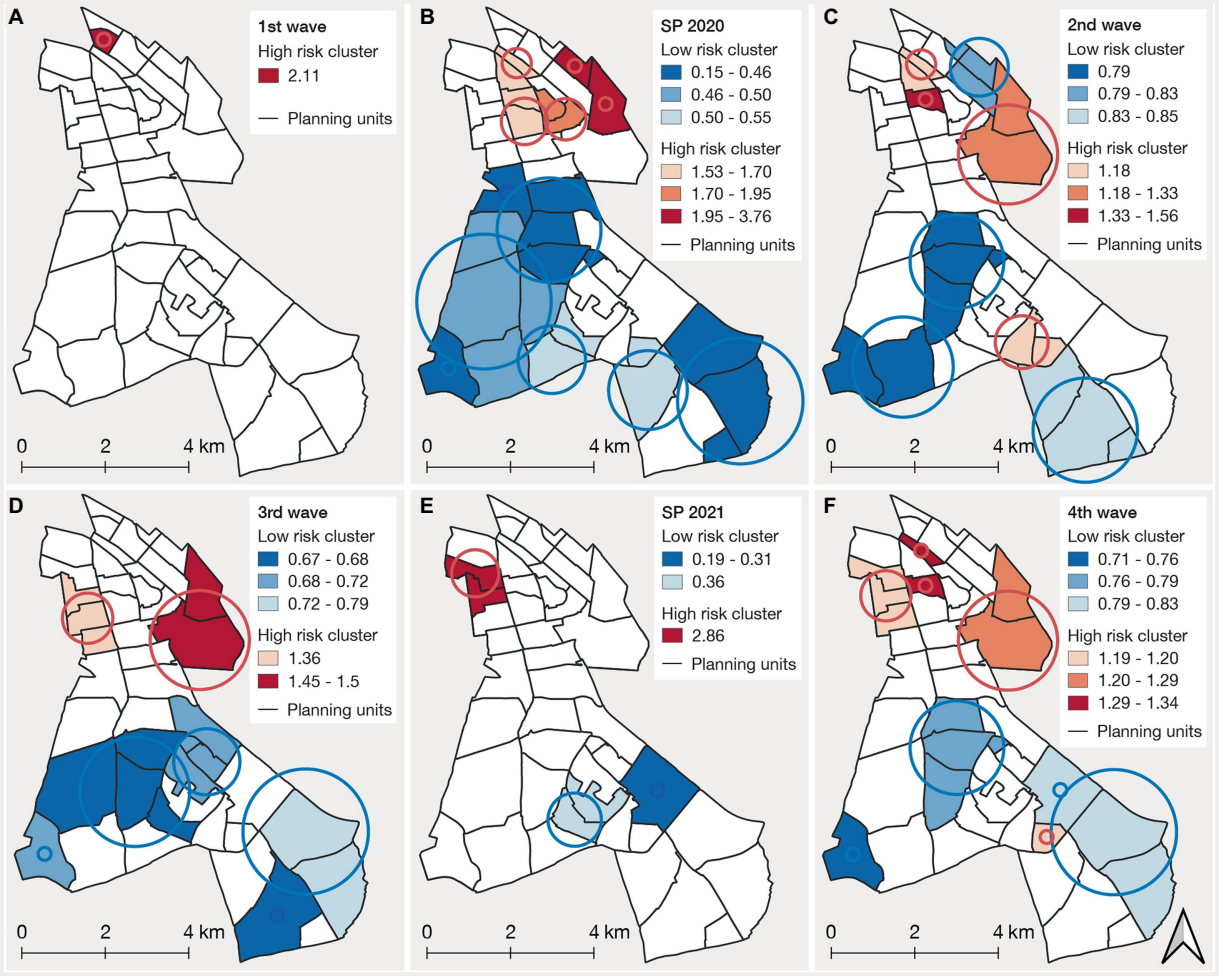
Spatial clusters of high-risk (red) and low-risk (blue) clusters of COVID-19 incidence across the six pandemic phases, including the four waves **(A,C,D,F)** and two summer plateaus (SP) **(B,E)** during the study period. The buffers represent the circular windows that were detected as significant spatial clusters. The size was determined by the centroids of the planning units located within the cluster.

The spatial distribution showed that cumulative incidence rates were heterogeneously spread over the district during the first wave (Figure 3A). Areas with high incidence were mainly in the southwestern and the northern part of the district, the lowest incidence being in the southeast. Only one significant high-risk cluster was detected for the first wave (Figure 4A). During the summer plateau of 2020, higher incidence was concentrated in the north, resulting in two high-risk clusters with a high relative risk value. In contrast, larger parts of the southeast and southwest exhibited comparably low incidence and low-risk clusters (Figures 3B,4B). The second wave was again characterized by a more heterogeneous distribution of cases. High incidence occurred partly in the northern and south-central parts of Neukölln, while planning units with low incidence were distributed variably across the whole district (Figure 3C). Clusters of high and low relative risk were both found in the northern and southern parts (Figure 4C). Planning units with high incidence reappeared in the third wave increasingly in the north, whereas areas with low incidence were observed more often in the south (Figure 3D). High-risk clusters were observed in the northeast and northwest, while large areas of the south were detected as low-risk clusters (Figure 4D). The summer plateau of 2021 and the fourth wave showed similar spatial patterns compared to the third wave, with high incidence and high-risk clusters more prevalent in the north and lower incidence and low-risk clusters more predominantly in the south (Figures 3E,F, 4E,F).

More details of the results can be explored *via* the web-based interactive visualization tool.

## 4. Discussion

The exploration of COVID-19 incidence on a small scale reveals differences in incidence across the pandemic phases, which are likely due to a set of influencing factors. The implementation of far-reaching interventions in particular seems to have had an effect on incidence in Neukölln. Other factors, such as the emergence of new virus variants, local events, and public holidays likely influenced incidence over time. Age group-specific incidence was found to have changed over the different pandemic phases. During the first and second waves, it was mainly the working population (18–44 and 45–65 years) and, to some extent, the elderly (> 65) who were affected more frequently, whereas the highest incidence was recorded among the young (< 18) during the third and fourth waves. It is likely that the implemented measures had different effects on age group-specific incidence. The spatial patterns across the pandemic phases revealed that incidence and cluster locations were heterogeneously distributed across the Neukölln district. The results also showed that the affectedness of areas can vary over time. Yet, certain areas in the north of the district showed high incidence and high-risk clusters over several phases of the pandemic, indicating a longer-lasting risk. Areas that were more often part of low-risk clusters were primarily concentrated in the south of the district.

### 4.1. Development of COVID-19 incidence and influencing factors

The pandemic phases exhibited great differences in terms of the total number of reported cases as well as in the dynamics of the rise and fall of cases. Each phase was characterized by a set of influencing factors that changed and interacted with one another constantly. For example, the two new virus variants, Alpha (B.1.1.7) and Delta (B.1.617.2), drastically changed incidence patterns during the third and fourth waves. Both variants were more transmissible (59, 60), which likely led to strong incidence increases at the beginning of the third and throughout the fourth wave in Neukölln. Interestingly, although the number of cases rose to unprecedented levels in the fourth wave, only a few social and physical distancing measures were in place at that time. One possible explanation might be the greater immunity among the population (either *via* previous infection or vaccination), which most often prevented severe illness (61, 62) and therefore reduced the need for stringent control measures.

Previous studies have also shown that various measures like social and physical distancing, mask-wearing, or testing strategies have been effective in reducing case numbers (2, 3, 52). However, the quantification of their direct impact on COVID-19 incidence is not always feasible, since interventions often came in packages and were influenced by a number of other factors (63). In our study, we are also hesitant to derive general statements. It may be observed that the far-reaching measures during the first, second, and third waves significantly decreased incidence in Neukölln, as also in Germany as a whole (14, 49). However, further analyses on neighborhood level, using, for example, spatiotemporal pandemic simulation models (64–67) could provide more insights to better understand the influence of policy interventions on case numbers.

Moreover, behavioral factors, e.g., the individual adaptations to daily routines to reduce potential exposure to the virus, might have influenced case transmissions as shown in previous studies (8, 68). Due to the absence of any individual behavioral data, we cannot derive any initial hypothesis on the impact of behavior on case numbers in this study.

It is relevant to note that the number of tests performed most likely influenced the case numbers and thereby the incidence. For example, the number of PCR tests doubled between early August and early November 2020 in Berlin (from 40,000 to 80,000 PCR tests per week) (69), which could also have contributed to the strong increases in the number of recorded cases at the beginning of the second wave. Conversely, test statistics revealed a substantial decrease in testing over vacation periods such as Christmas or Easter, which may also be reflected in the short-term decreases in cases over these periods. The impact of testing on the occurrence of cases has also been shown in other regions and in other pandemic phases (14, 70). Still, frequent and large-scale rapid testing was evidently an effective means of decreasing case numbers in Germany during the third pandemic wave (71). The introduction of rapid antigen tests has made it possible to identify cases that would otherwise remain undetected and to isolate infected individuals in time before the infection was passed on to others.

The exploration of cases also showed that not only the numerous measures in Berlin as a whole (Supplementary Figure 1) but also local events and measures influenced case development. This can be especially observed with overall low incidences. In Neukölln, the influence of local events was illustrated by the major outbreak in a residential complex during the summer plateau of 2020. A sharp increase in incidence occurred, which quickly leveled off again after quarantine and testing measures were implemented locally. This demonstrates how important it is to understand local events and interventions when interpreting changes in incidence in a study area.

Finally, the results showed that seasonality may have an effect in slowing the spread of infections. Incidence was substantially lower in the summer compared to the winter months in Neukölln, which is in line with previous research that found that infectiousness increased during winter, owing to more frequent indoor gatherings and weakened immune systems (10, 11, 71).

### 4.2. Age group-specific differences in incidence

Our findings show that single measures focused specifically on the reduction of transmissions within certain age groups. Measures relating to schools and childcare centers were mainly implemented to reduce incidence among the young (<18 years). For Neukölln, school and childcare center closures during the first and second waves were consistent with an incidence reduction among young people. Similarly, however, the number of cases decreased among the young during school holidays (e.g., summer holidays 2020 and 2021, autumn holidays 2021). Testing in schools may also have influenced the number of cases in this population. For example, schoolchildren had to test themselves several times a week after the summer holidays in 2021, which also likely contributed to an increase in the incidence of recorded cases among the young at the beginning of August 2021.

In contrast, the vaccination campaign was initially a measure specifically targeted at the elderly. During the third wave, when the elderly were prioritized for vaccination, the number of cases among the elderly was, for the first time, significantly lower than in other age groups. This trend continued in the fourth wave, with the lowest incidence being found among the elderly. The high rates of vaccination of the elderly in Berlin during the third and fourth waves (72) are likely to have prevented numerous cases among this group. Still, it has been shown for Germany that, especially in the 6 months after the start of the vaccination campaign in December 2021, the effects of vaccinations on the total number of infections were probably minimal, as the vaccination rates of the whole population were rather low compared to other countries (71, 73).

Further, the results highlight that, even when certain measures target all age groups similarly, they still can lead to different incidence between age groups. For example, after the partial lockdown in mid-November 2020 was imposed, incidence among the young and working population decreased, while it increased among the elderly. The same development was also identified in other regions in Germany, where incidence among the elderly revealed a different infection dynamic than other age groups after the implementation of measures in mid-November (14). It was hypothesized, based on the findings of Thurner et al. (74) that infection dynamics among the elderly might be characterized by a linear or diffuse growth dynamic, owing to lower connectivity in their social networks. In contrast, the young and working populations may show higher connectivity in their networks, resulting in an exponential growth dynamic, where in particular super-spreading events may play a major role. Strict measures for contact reduction mainly prevent exponential growth but have only little effect on networks with low connectivity (75, 76). This observation may also apply to Neukölln. On the one hand, it may be observed that far-reaching measures had a major impact on the young and working population by preventing further exponential growth. On the other hand, the measures showed only minor effects on incidence among the elderly, which could lead to the hypothesis that, in Neukölln, too, incidence among this group tends to exhibit a linear or diffuse growth dynamic.

### 4.3. Spatiotemporal patterns and clusters

The results of our spatial analysis highlight that the distribution of COVID-19 incidence is most often heterogeneous, with changing spatial patterns across the different pandemic phases. Other intra-urban studies have shown similarly heterogeneous patterns, with spatial distributions also changing over the course of different phases (25, 26, 35). A number of studies have tried to understand how these spatial patterns emerge on a small scale by analyzing the relationship between COVID-19 cases and socioeconomic and demographic characteristics or the degree to which an area is built up. These studies indicated that socioeconomically disadvantaged areas were more susceptible to the spread of COVID-19 on a small scale. COVID-19 cases were, for example, negatively related to income (25, 26, 77) or indices based on socioeconomic data (78–82) and positively related to population density, average household size (77, 83, 84), and the proportion of young people in the population (26, 77). These analyses are still pending for Neukölln and no general statements can be made on the basis of our initial exploration. However, our results allow us to draw initial hypotheses about the association between the abovementioned characteristics of the planning units and COVID-19 cases. The detection of clusters during the second, third, and fourth waves reveals that high-risk areas were located in neighborhoods with a low to very low socioeconomic status index, while low-risk areas were mainly characterized by a medium socioeconomic status index. Further, high incidence was mostly found in areas with dense housing and a high population density. On the contrary, the less affected areas were mainly situated in areas with dispersed housing and a lower population density. With regard to the age distribution in the district, the results showed that low-risk clusters were concentrated in areas with a high percentage of elderly people. These initial visual comparisons may indicate an association between factors such as socioeconomic status, residential density, or age and COVID-19 incidence for Neukölln. However, such exploratory results require examination in further in-depth modeling analyses as for example conducted (77, 85).

Initial explanations may also be given for the changes in the patterns over time. We observed that in Neukölln, the first wave differed in its spatial patterns compared to the consecutive phases. Similar patterns were also found at the national level (86, 87) and in other intra-urban settings (26). One possible explanation might relate to the varying effects of the pandemic on different types of employment. During the first wave, higher incidence might be attributed to employed people with higher mobility (e.g., business travel, transregional commuting, or holidays), whereas high incidence during the consecutive waves could have occurred mainly among people in less advantaged occupations, who were not able to work from home (86). In addition, it has been shown that the impact of far-reaching measures can affect socioeconomic groups differently (88), which also could have resulted in different spatial patterns over time. In Neukölln, for example, we see that, during the second and third wave, when extensive control measures were implemented, socioeconomically disadvantaged areas experienced a higher risk compared to other areas. Still, analyses with a higher temporal resolution are needed to investigate this relationship in more detail.

### 4.4. Limitations

Our study is subject to some limitations. First, we used reported cases, which have been shown to deviate from the number of true cases, as shown in previous studies for Germany (89, 90). Test capacities and test strategies add a bias to the reported case data. Not only did the test capacity increase over time in Germany, but there were also changes in the testing strategy and availability and accessibility of tests. At the beginning of the pandemic, only symptomatic persons were tested, which likely led to many asymptomatic cases not being detected. Later on, travel returnees or contacts of positive-tested persons were also tested, leading to more actual cases being detected. However, reported cases are still one of the main indicators to trace transmissions in disease surveillance systems, and have been proven to be useful in other studies to explore the relationship between incidence and interventions (3). Most importantly, there are no other comprehensive data available at the neighborhood level (e.g., testing capacities, tests conducted, or severity of the disease).

The second limitation is due to the aggregated analysis of COVID-19 cases at the planning unit level, which influences the results by the different shapes and sizes of the underlying unit. Thus, our analysis is—like other spatial analyses—subject to the modifiable areal unit problem (MAUP), which describes the bias when aggregating spatial point data into larger spatial features (91). However, we believe that the planning units are a suitable unit of analysis because they reveal a high spatial resolution and are, unlike zip codes, quite homogeneous within themselves in terms of built structures and socio-demographic characteristics. This enables us to identify high-risk areas on a small scale and thus to provide support for developing locally specific measures that target vulnerable locations and groups.

## 5. Conclusions and outlook

In our study, we aimed to fill the gap in COVID-19 research at the neighborhood level by exploring the development of COVID-19 incidence and by identifying spatiotemporal patterns over time, using the example of the Berlin district Neukölln. We highlight that a number of factors have influenced the development of COVID-19 incidence and that such factors have changed over the course of the pandemic phases. We identified the locations of disease clusters over time and provided information about high-risk areas, which were affected particularly often. It is important to note that we cannot draw any general conclusions from our explanatory study regarding the specific impact of influencing factors or the relationship between characteristics of a neighborhood and COVID-19 incidences. Still, the results of our descriptive epidemiological study provide new insights for public health authorities, community planners, and policymakers about the mechanisms of COVID-19 transmission on a small scale. These insights are crucial for guiding decision-makers in implementing mitigation strategies. Future research should concentrate on detailed analyses of the relationship between neighborhood characteristics (e.g., socioeconomic, environmental, the proportion of built-up areas) and COVID-19 incidence at the neighborhood level. To date, the ways in which these relationships have changed at the neighborhood level over the course of the pandemic phases have remained largely unexplored. Also, we suggest that further studies should investigate the association between measures and spatial patterns at a high temporal resolution to better understand how measures have impacted locations and population groups differently.

For future studies, we believe that collaborations with local health authorities are essential, since they can not only provide fine-grained data but also give important insights into the pandemic situation, which are crucial to understanding transmission patterns on a small scale. Further, we make a plea for accessible datasets, since they uniquely enable research that can provide important insights for this and future pandemics.

## Data availability statement

The data analyzed in this study is subject to the following licenses/restrictions: The data that support the findings of this study were obtained from the Department of Public Health Neukölln, but restrictions apply to the availability of these data, which were used solely for the current study, and so are not publicly available. Data are available from the authors upon reasonable request and only with permission of the Department of Public Health Neukölln. Requests to access these datasets should be directed to: tillman.schmitz@hu-berlin.de.

## Ethics statement

The studies involving human participants were reviewed and approved by the Ethic committee of the Faculty of Mathematics and Natural Sciences of Humboldt University Berlin. Written informed consent for participation was not required for this study in accordance with the national legislation and the institutional requirements.

## Author contributions

TS and TL: study design. TS, CL, and GM: data preparation. TS: data analysis. TS and TL: writing of the manuscript. TS and GM: creation of figures. TS, TL, CL, JB, GM, AR, and NS: manuscript revision. All authors contributed to the article and approved the submitted version.

## Funding

This study was funded by the Deutsche Forschungsgemeinschaft (DFG, German Research Foundation; 492361591) and the Open Access Publication Fund of Humboldt-Universität zu Berlin.

## Conflict of interest

The authors declare that the research was conducted in the absence of any commercial or financial relationships that could be construed as a potential conflict of interest.

## Publisher’s note

All claims expressed in this article are solely those of the authors and do not necessarily represent those of their affiliated organizations, or those of the publisher, the editors and the reviewers. Any product that may be evaluated in this article, or claim that may be made by its manufacturer, is not guaranteed or endorsed by the publisher.

## Footnotes

^1^ https://spopt.geographie.hu-berlin.de/covid/
