# Supplementary material for: Exploration of the COVID-19 pandemic at the neighborhood level in an intra-urban setting
Source: Front Public Health. 2023 Apr 13;11:1128452. doi: 10.3389/fpubh.2023.1128452 (PMC10133460; doi:10.3389/fpubh.2023.1128452)
Supplement: Supplementary file 1 [file Data_Sheet_1.docx]

Supplementary Material

# Supplementary Figures and Tables

## Supplementary Tables

# Supplementary Table 1. Parameter settings for the spatiotemporal pattern analysis

| **Parameter** | **Parameter setting** | **Reasoning** |
| --- | --- | --- |
| Type of Analysis | Retrospective analysis: purely spatial | We decided for a purely spatial analysis since we were interested in the spatial distribution of clusters for the entire period of each phase and not for the exact time period of cluster occurrence. Thus, the analysis was done only once for each phase for the fixed geographical region ignoring the time of cases. |
| Probability Model | Discrete Scan Statistics: Poisson | We used the discrete Poisson model to account for the background population within each planning unit. This probability model is used when the background population reflects a certain risk and the cases are included as part of the population count (compared to the Bernoulli model where the input data set also includes control data). |
| Maximum spatial cluster size | 7.5 % of the population at risk | We compared different maximum spatial cluster sizes and then iteratively decided on the maximum spatial cluster size of 7.5% of the population at risk to avoid large clusters and concentrate on small clusters. By using a smaller value (compared to the 50% default value), the detection of small and homogeneous clusters is emphasized (1). |
| Minimum number of cases | 5 | To avoid clusters with only a few cases, we decided to set the minimum number of cases to five similarly to other studies (2,3). |
| Scan for Areas with | High or low rates | We decided to scan simultaneously for areas with either high or low rates. On the one hand, we were interested in areas depicting high risk to locate vulnerable neighborhood where mitigation measures can be implemented in a more targeted way. On the other hand, areas with lower risk may indicate more resilient neighborhood and provide insight into why neighborhood were better prepared and protected during the different pandemic phases. |
| Inference | Default p-value method with maximum number of Monte Carlo replications = 999 | We used the default p-value method with the maximum number of Monte Carlo replications set to 999 in accordance to other COVID-19 studies using spatial scan statistics (3,4). |
| Criteria for Reporting hierarchical clusters | No geographical overlap | To avoid secondary clusters that overlap with a previously reported cluster, we set the criteria for reporting hierarchical clusters to no geographical overlap. |

# Supplementary Table 2. Detailed description of public health and social measures and further events in Berlin and Neukölln

| **Month  (week)** | **Measure or event** | **Detailed description** | **Source** |
| --- | --- | --- | --- |
| **2020** | | | |
| March (11/12) | First far-reaching social and physical distancing measures | - Stay-at-home order for all residents except for essential activities (e.g., food shopping, traveling to work…) - Only essential private gatherings are allowed if the number of people is <10 (e.g., funerals…) - Public gatherings with > 20 people only with permission of the administration authority - Closure of businesses, institutions, and services (e.g., retail, clubs, bars, cinemas, cultural centers, conferences, museums, concerts, restaurants, hotels, …) - 14-day quarantine order for travelers entering the country (exceptions listed in the 2^nd^ revision of the regulation) | (5–7) |
| March (11) | School & childcare center closure | - Partial closure of schools to children, students, teachers, and staff - Teachers and staff may remain to provide lessons to students/ care for the children of essential workers - *Target*: childcare centers, primary schools, secondary schools | (5) |
| April (15) | Relaxation of measures | - e.g., lifting of the stay-at-home order, outdoor sports allowed again, tourist overnight stays with hygiene requirements possible again, relaxed limits on numbers for private and public gatherings, gradual opening of cultural and educational institutions… | (8–10) |
| April (16) | Childcare center opening | - Opening of childcare centers under certain conditions (e.g., cohort model. Cohorts are groups of children and staff who interact mostly only with each other. The goal is to limit the number of people anyone is exposed to) | (8) |
| April (17) | Face mask requirements | - Requirement to wear a face mask, successively also in further closed settings (e.g., in schools, on public transportation, in retail, restaurants, services…) | (9,11–13) |
| May (18) | School opening | - Opening of schools under certain conditions (e.g., graduated opening according to school type, school levels, grades, and courses of education. Further details are determined by the authorities responsible for the school system) | (11) |
| June (24) | Active case detection & quarantinization | - COVID-19 outbreak in a residential complex in north Neukölln - Extensive active case detection and quarantinization implemented by the health department | (14) |
| September (39) | General quarantine rule | - Previously, all persons tested positive had to be informed about quarantinization directly by the health department which led to a high workload resulting in occasional delays. - With the new general quarantine rule, quarantine no longer needed to be ordered by the health department (passive case detection) - Active case detection only in facilities | (15) |
| October (42) | New testing strategy in patient care facilities | - As of October 15, individuals in patient care facilities, such as hospitals and nursing homes, can be tested for coronavirus using rapid antigen tests (administered by medical doctors or in pharmacies) - *Target*: Employees, visitors, and residents or persons in care | (16) |
| November (44) | Partial lockdown | - Reintroduction of far-reaching social and physical distancing measures - Private gatherings in public places only with members of one’s own household (max. 10 people, violations are sanctioned) - Universities must provide digital teaching virtually - Working from home must be ensured - Closure of businesses, (recreational) institutions, and services (cinema, cultural centers, museums, concerts, restaurants …) - Successive intensification of restrictions on contact (e.g., private gatherings in public places only with members of one’s own household + one additional person, max. 5 people) - Christmas Eve: household members + four additional persons; New Year’s Eve: cancellation of private and public gatherings - Extension of restrictions on travelers entering the country from region where there is a variant of concern (VOC) | (17–20) |
| December (50) | Extended lockdown | - Extension of the social and physical distancing measures - Successively, as of January 20, requirement to wear a medical mask in public transportation and closed settings and other measures - Private gatherings only with household members + one additional person | (21) |
| December (50) | School & childcare center closure | - Partial closure of schools to children, students, teachers, and staff - Teachers and staff may remain to provide lessons to students/ care for the children of essential workers - *Target*: childcare centers, primary schools, secondary schools | (22) |
| December (52) | Vaccination campaign with prioritization strategy | - Beginning of the vaccination campaign - Prioritization by age group, nursing home residents, medical facility staff, medical indication, persons with precarious working and/or living conditions | (23,24) |
| **2021** | | | |
| March (9) | Childcare center opening | - Opening of childcare centers under certain conditions (e.g., cohort model. Cohorts are groups of children and staff who interact mostly only with each other. The goal is to limit the number of people anyone is exposed to) | (25) |
| March (9) | Authorization of rapid antigen tests | - First authorization of at-home self-testing with rapid antigen tests by laypersons - In addition, one test per person per week was made available free of charge at test centers | (26) |
| March (10) | COVID-19 certificate regulation for businesses and services | - Presentation of a certificate of a negative COVID-19 test for businesses and services (close-contact services, successively also in retail and shopping malls, events, restaurants...) - As of April 28, vaccinated and recovered persons no longer need to provide proof of a negative test in order to gain access to many places | (27) |
| April (13) | Testing strategy for employees | - Introduction of a testing policy under which employees are offered free antigen testing regularly, at least once or twice a week, organized by the employer | (28,29) |
| May (17) | Bundesnotbremse (literally ‘federal emergency brake’) | - New strategy of applying restrictions according to local incidence, e.g., if the 7-day incidence per 100,000 inhabitants rises to over 100 on three consecutive days, stricter rules come into force again - Measures are defined by the federal government and are no longer the responsibility of the federal states | (30) |
| May (21) | School opening | - Opening of schools under certain conditions (e.g., graduated opening according to school types, school levels, grades, and courses of education) | (31) |
| June (22) | Relaxation of measures | - E.g., relaxation of contact and private/public gathering and for public event restrictions. Facilities for dinging out now allowed to open again for guests… | (32) |
| June (22) | New case management software | - Migration to the nationally used software SORMAS (Surveillance Outbreak Response Management and Analysis System) for case and contact management - Unification of the previously separately managed databases for case and contact tracing | (33) |
| July (27) | Lifting of vaccination prioritization | - As of June 7, COVID-19 vaccinations were available to everyone without prioritization | (34) |
| August (34) | 3G regulations | - New strategy for access to various facilities under certain conditions - 3G stands for *geimpft, genesen, getestet* [vaccinated, recovered, tested] - Applies, for example, in hospitals, nursing homes, indoor catering, indoor events, close-contact services, … - Other businesses that were fully closed before may open under 2G conditions | (35,36) |
| September (37) | 2G regulations | - 2G stands for *geimpft, genesen* [vaccinated, recovered] - For almost all the facilities, businesses, institutions, and services listed above under 3G conditions - Gradually further differentiations according to 3G, 3G+, 2G, 2G+   3G (vaccinated **or** recovered **or** tested with rapid antigen test)  3G+ (vaccinated **or** recovered **or** tested with PCR test)  2G (vaccinated **or** recovered)  2G + (vaccinated **or** recovered **and** tested with rapid antigen test **or** booster vaccination) | (37) |

## Supplementary Figures

**Measure-index:** The measure-index (also called measure-stringency-index) was created to approximate the stringency of measures in Germany at the state and county level over time (38). The index is based on the Oxford-Stringency-Index (39) and is calculated as follows: At first, official publications on COVID-19 regulations were collected at the state and county level. The measures published in the regulations were then classified into different main and subcategories. Within each main category, subcategories were ordinally sorted by stringency and assigned a numerical value (e.g., measures comprising the complete closures of facilities will be given a higher value than measures that limit the maximum number of people in a facility). Since there is a different number of subcategories per main category, all assigned numerical values within a main category are subsequently scaled from 1 to 100. The highest value within a main category is then determined on a daily basis. To derive the final measure-index, a mean value across the highest values of the main categories is calculated.
We used the index to approximate the stringency and duration of measures. Above that, we used the index to identify points in time that have caused a major change in measures, either in terms of the introduction of far-reaching measures or the reduction of measures.

**Supplementary Figure 1.** Berlin-wide COVID-19 regulations and the measure-index, across pandemic phases, including the four waves and two summer plateaus (SP) during the study period. The regulations that have been published/entered into force at the time of the index change are marked in red. Text structure: regulation document abbreviation; date published; date entered into force. The regulation documents can be consulted in the archive of the Berlin House of Representatives (40).

# References

1. Chen J, Roth RE, Naito AT, Lengerich EJ, MacEachren AM. Geovisual analytics to enhance spatial scan statistic interpretation: An analysis of U.S. cervical cancer mortality. *Int J Health Geogr* (2008) 7:1–18. doi: 10.1186/1476-072X-7-57/COMMENTS

2. Greene SK, Peterson ER, Balan D, Jones L, Culp GM, Fine AD, Kulldorff M. Detecting COVID-19 Clusters at High Spatiotemporal Resolution, New York City, New York, USA, June–July 2020. *Emerg Infect Dis* (2021) 27: doi: 10.3201/eid2705.203583

3. Siljander M, Uusitalo R, Pellikka P, Isosomppi S, Vapalahti O. Spatiotemporal clustering patterns and sociodemographic determinants of COVID-19 (SARS-CoV-2) infections in Helsinki, Finland. *Spat Spatiotemporal Epidemiol* (2022) 41: doi: 10.1016/j.sste.2022.100493

4. Ladoy A, Opota O, Carron PN, Guessous I, Vuilleumier S, Joost S, Greub G. Size and duration of COVID-19 clusters go along with a high SARS-CoV-2 viral load: A spatio-temporal investigation in Vaud state, Switzerland. *Science of the Total Environment* (2021) 787: doi: 10.1016/j.scitotenv.2021.147483

5. Berlin Senate. Verordnung über erforderliche Maßnahmen zur Eindämmung der Ausbreitung des neuartigen Coronavirus SARS-CoV-2 in Berlin vom 17.03.2020. Berlin (2020). https://pardok.parlament-berlin.de/starweb/adis/citat/VT/18/vo/vo18-207.pdf [Accessed November 22, 2022]

6. Berlin Senate. Verordnung über erforderliche Maßnahmen zur Eindämmung der Ausbreitung des neuartigen Coronavirus SARS-CoV-2 in Berlin vom 22.03.2020. Berlin (2020). https://pardok.parlament-berlin.de/starweb/adis/citat/VT/18/vo/vo18-210.pdf [Accessed November 22, 2022]

7. Berlin Senate. Zweite Verordnung zur Änderung der SARS-CoV-2-Eindämmungsmaßnahmenverordnung vom 09.04.2020. Berlin (2020). https://pardok.parlament-berlin.de/starweb/adis/citat/VT/18/vo/vo18-212.pdf [Accessed November 22, 2022]

8. Berlin Senate. Dritte Verordnung zur Änderung der SARS-CoV-2-Eindämmungsmaßnahmenverordnung vom 16.04.2020. Berlin (2020). https://pardok.parlament-berlin.de/starweb/adis/citat/VT/18/vo/vo18-213.pdf [Accessed November 22, 2022]

9. Berlin Senate. Dritte Verordnung zur Änderung der SARS-CoV-2-Infektionsschutzverordnung vom 04.08.2020. Berlin (2020). https://pardok.parlament-berlin.de/starweb/adis/citat/VT/18/vo/vo18-245.pdf [Accessed November 29, 2022]

10. Senat von Berlin. SARS-CoV-2-Infektionsschutzverordnung vom 23.06.2020. Berlin (2020). https://pardok.parlament-berlin.de/starweb/adis/citat/VT/18/vo/vo18-230.pdf [Accessed November 30, 2022]

11. Berlin Senate. Vierte Verordnung zur Änderung der SARS-CoV-2-Eindämmungsmaßnahmenverordnung vom 21.04.2020. Berlin (2020). https://pardok.parlament-berlin.de/starweb/adis/citat/VT/18/vo/vo18-214.pdf [Accessed November 22, 2022]

12. Berlin Senate. Sechste Verordnung zur Änderung der SARS-CoV-2-Eindämmungsmaßnahmenverordnung vom 07.05.2020. Berlin (2020). https://pardok.parlament-berlin.de/starweb/adis/citat/VT/18/vo/vo18-216.pdf [Accessed November 22, 2022]

13. Berlin Senate. Fünfte Verordnung zur Änderung der SARS-CoV-2-Eindämmungsmaßnahmenverordnung vom 28.04.2020. Berlin (2020). https://pardok.parlament-berlin.de/starweb/adis/citat/VT/18/vo/vo18-203.pdf [Accessed November 22, 2022]

14. rbb. Wohnblock in Berlin-Neukölln unter Corona-Quarantäne. (2020) https://www.rbb24.de/panorama/thema/2020/coronavirus/beitraege_neu/2020/06/berlin-neukoelln-corona-ausbruch-wohnblock-quarantaene-schulen.html [Accessed November 22, 2022]

15. District Office Berlin-Neukölln. Neue Allgemeinverfügung zur Quarantäne in Neukölln. (2020) https://www.berlin.de/ba-neukoelln/aktuelles/pressemitteilungen/2020/pressemitteilung.1002091.php [Accessed November 22, 2022]

16. Federal Ministry of Health. Coronavirus-Pandemie: Was geschah wann? *2022* https://www.bundesgesundheitsministerium.de/coronavirus/chronik-coronavirus.html [Accessed November 22, 2022]

17. MPK-Beschluss vom 28.10.2020. (2020) https://www.bundesregierung.de/resource/blob/975232/1805024/5353edede6c0125ebe5b5166504dfd79/2020-10-28-mpk-beschluss-corona-data.pdf [Accessed November 22, 2022]

18. MPK-Beschluss vom 25.11.2020. (2020) https://www.bundesregierung.de/resource/blob/975226/1820174/fd9794fa8b8e0ec555f005677509c242/2020-11-25-mpk-beschluss-data.pdf?download=1 [Accessed November 22, 2022]

19. MPK-Beschluss vom 13.12.2020. (2020) https://www.bundesregierung.de/resource/blob/975226/1827366/69441fb68435a7199b3d3a89bff2c0e6/2020-12-13-beschluss-mpk-data.pdf [Accessed November 22, 2022]

20. MPK-Beschluss vom 05.01.2021. (2021) https://www.bundesregierung.de/resource/blob/975226/1834306/75346aa9bba1050fec8025b18a4bb1a3/2021-01-05-beschluss-mpk-data.pdf [Accessed November 22, 2022]

21. MPK-Beschluss vom 19.01.2021. (2021) https://www.bundesregierung.de/resource/blob/974430/1840868/1c68fcd2008b53cf12691162bf20626f/2021-01-19-mpk-data.pdf?download=1 [Accessed November 22, 2022]

22. Berlin Senate. Verordnung zur Neufassung der Berliner Vorschriften zum Schutz vor Infektionen mit dem Coronavirus SARS-CoV-2 vom 14.12.2020. Berlin (2020). https://pardok.parlament-berlin.de/starweb/adis/citat/VT/18/vo/vo18-283.pdf [Accessed November 22, 2022]

23. Robert-Koch-Institut. Stufenplan der STIKO zur Priorisierung der COVID-19-Impfung. (2021) https://www.rki.de/DE/Content/Infekt/Impfen/ImpfungenAZ/COVID-19/Stufenplan.pdf?__blob=publicationFile [Accessed November 22, 2022]

24. rbb. Corona-Impfungen in Berlin haben begonnen. (2020) https://www.rbb24.de/panorama/thema/2020/coronavirus/beitraege_neu/2020/12/berlin-corona-impfung-beginn-pflegeheim-steglitz-gertrud-haase.html [Accessed November 22, 2022]

25. SenBJF. Kitas ab 17. Mai wieder für alle Berliner Familien und Kinder geöffnet. (2021). https://www.berlin.de/sen/bjf/service/presse/pressearchiv-2021/pressemitteilung.1084365.php [Accessed November 22, 2022]

26. Federal Ministry of Health. Erste Zulassungen für Selbsttests. (2021) https://www.bundesregierung.de/breg-de/themen/coronavirus/zulassung-schnell-test-1861354 [Accessed November 22, 2022]

27. Berlin Senate. Zweite Verordnung über erforderliche Maßnahmen zum Schutz der Bevölkerung vor Infektionen mit dem Coronavirus SARS-CoV-2 vom 04.03.2021. Berlin (2021). https://pardok.parlament-berlin.de/starweb/adis/citat/VT/18/vo/vo18-325.pdf [Accessed November 22, 2022]

28. Berlin Senate. Erste Verordnung zur Änderung der Zweiten SARS-CoV-2-Infektionsschutzmaßnahmenverordnung vom 23.03.2021. Berlin (2021). https://pardok.parlament-berlin.de/starweb/adis/citat/VT/18/vo/vo18-335.pdf [Accessed November 22, 2022]

29. Berlin Senate. Zweite Verordnung zur Änderung der Zweiten SARS-CoV-2-Infektionsschutzmaßnahmenverordnung vom 27.03.2021. Berlin (2021). https://pardok.parlament-berlin.de/starweb/adis/citat/VT/18/vo/vo18-336.pdf [Accessed November 22, 2022]

30. MPK-Beschluss vom 03.03.2021. (2021) https://www.bundesregierung.de/resource/blob/974430/1872054/66dba48b5b63d8817615d11edaaed849/2021-03-03-mpk-data.pdf?download=1 [Accessed November 22, 2022]

31. Senat von Berlin. Zwölfte Verordnung zur Änderung der Schul-Hygiene-Covid-19-Verordnung. Berlin (2021). https://pardok.parlament-berlin.de/starweb/adis/citat/VT/18/vo/vo18-369.pdf [Accessed November 22, 2022]

32. Senat von Berlin. Achte Verordnung zur Änderung der Zweiten SARS-CoV-2-Infektionsschutzmaßnahmenverordnung vom 01.06.2021. Berlin (2021). https://pardok.parlament-berlin.de/starweb/adis/citat/VT/18/vo/vo18-366.pdf [Accessed November 30, 2022]

33. Helmholtz-Zentrum für Infektionsforschung. SORMAS ÖGD COVID-19. (2021) https://www.sormas-oegd.de/ [Accessed November 29, 2022]

34. Federal Ministry of Health. Impf-Priorisierung aufgehoben. (2021) https://www.bundesregierung.de/breg-de/themen/coronavirus/corona-impfung-priorisierung-entfaellt-1914756 [Accessed November 22, 2022]

35. MPK-Beschluss vom 10.08.2021. https://www.bundesregierung.de/resource/blob/974430/1949532/d3f1da493b643492b6313e8e6ac64966/2021-08-10-mpk-data.pdf?download=1 [Accessed November 22, 2022]

36. Berlin Senate. Vierte Verordnung zur Änderung der Dritten SARS-CoV-2-Infektionsschutzmaßnahmenverordnung vom 17.08.2021. Berlin (2021). https://pardok.parlament-berlin.de/starweb/adis/citat/VT/18/vo/vo18-394.pdf [Accessed November 22, 2022]

37. Berlin Senate. Achte Verordnung zur Änderung der Dritten SARS-CoV-2-Infektionsschutzmaßnahmenverordnung vom 05.10.2021. Berlin (2021). https://pardok.parlament-berlin.de/starweb/adis/citat/VT/18/vo/vo18-412.pdf [Accessed November 22, 2022]

38. infas 360. Maßnahmenindex Bundesländer pro Tag . (2022) https://www.corona-datenplattform.de/dataset/massnahmenindex_bundeslaender_pro_tag [Accessed November 1, 2022]

39. Hale T, Angrist N, Goldszmidt R, Kira B, Petherick A, Phillips T, Webster S, Cameron-Blake E, Hallas L, Majumdar S, et al. A global panel database of pandemic policies (Oxford COVID-19 Government Response Tracker). *Nat Hum Behav* (2021) 5:529–538. doi: 10.1038/s41562-021-01079-8

40. Abgeordnetenhaus Berlin. Archiv. (2022) https://pardok.parlament-berlin.de/portala/browse.tt.html [Accessed November 30, 2022]
